# Systemic Effects of Oral Antibiotics in Mice: A Combined Physiological and Molecular Approach

**DOI:** 10.3390/biom16030409

**Published:** 2026-03-10

**Authors:** Ilir Mazreku, Aulon Kasolli, Zana Gerxhaliu, Melek Smaili, Avni Berisha, Savaş Kaya, Alejandro Morales-Bayuelo

**Affiliations:** 1 Department of Biology, Faculty of Math and Natural Science, University of Prishtina, 10000 Pristina, Kosovo; ilir.mazreku@uni-pr.edu (I.M.); aulon.kasolli@student.uni-pr.edu (A.K.); zana.gerxhaliu@student.uni-pr.edu (Z.G.); melek.smaili@student.uni-pr.edu (M.S.); 2 Department of Chemistry, Faculty of Math and Natural Science, University of Prishtina, 10000 Pristina, Kosovo; 3 Department of Chemistry, Faculty of Science, Sivas Cumhuriyet University, 58140 Sivas, Turkey; savaskaya@cumhuriyet.edu.tr; 4 Grupo Genoma, Escuela de Medicina, Universidad del Sinú, Seccional Cartagena, Cartagena 230001, Colombia

**Keywords:** amoxicillin, tetracycline, food intake, antibiotics, oral treatment

## Abstract

This study investigates the systemic effects of amoxicillin and tetracycline on healthy Mus musculus (Swiss albino) mice, focusing on food intake, body weight, and hematological parameters. Over a 14-day oral treatment period, both antibiotics significantly reduced weight gain and food efficiency, with sex-specific variations: tetracycline had stronger metabolic effects in males, while amoxicillin was more impactful in females. To explore underlying mechanisms, molecular docking and MM-GBSA analyses were performed on PPAR-γ and TLR2–TIRAP complexes. Both antibiotics showed negligible binding to PPAR-γ, suggesting their metabolic effects are not receptor-mediated. In contrast, tetracycline exhibited strong and stable binding to TLR2 (ΔG_bind_ = −27.87 kcal/mol), supported by extensive hydrogen bonding, implying potential immunomodulatory action. These findings suggest that antibiotic-induced metabolic and immune alterations are more likely driven by microbiota disruption and innate immune signaling, rather than direct metabolic receptor engagement.

## 1. Introduction

In both human and veterinary medicine, antibiotics are among the most often prescribed medications for the treatment of bacterial infections. But their widespread and unchecked use has caused worries about the emergence of antibiotic resistance [1]. In addition to this well-established problem, their effects on fundamental physiological functions in healthy organisms, like appetite and food intake, are a lesser-studied but no less significant factor [2].

Antibiotics are low-molecular-weight microbial metabolites that can stop microorganisms from growing. This growth inhibition or suppression may be temporary or permanent. Antibiotics have a complex chemical structure that may contain carbon, hydrogen, oxygen, and nitrogen, but in some cases, they can also include sulfur, phosphorus, or atoms from the halogen group [3]. Based on the arrangement of these atoms in their chemical structure, they are classified into different groups that include: beta-lactams, macrolides, tetracyclines, quinolones, aminoglycosides, glycopeptides, oxazolidinones, and sulfonamides [4].

For this research, amoxicillin and tetracycline were chosen, because of their different modes of action and widespread use in both human and veterinary medicine as a broad-spectrum antibiotic. More than 50% of the cases where these antibiotics are prescribed could be replaced by a narrow spectrum antibiotic [5]. The broad-spectrum antibiotics have broader effects on gut microbiota, and are better candidates for combined studies. Amoxicillin, being a β-lactam antibiotic, primarily targets bacterial cell wall synthesis, leading to cell lysis and death of bacteria [6]. In contrast, tetracycline is a broad-spectrum antibiotic that inhibits protein synthesis by binding to the 30S ribosomal subunit, preventing bacterial growth and replication as a result [7]. These fundamental differences in their modes of action provide an opportunity to explore and compare their diverse effects on these physiological parameters.

Although these antibiotics are mainly known for their role in fighting bacterial infections, they may also lead to various side effects on the host organism. After use, amoxicillin may cause side effects such as nausea, vomiting, diarrhea, tooth discoloration, swelling of the face, throat or tongue, difficulty breathing or swallowing, as well as extreme fatigue [8]. Similarly, tetracycline can lead to side effects including skin redness or blistering, fever, body aches, yellowing of the skin, loss of appetite, stomach discomfort, white patches inside the mouth, swollen tongue and difficulty swallowing [9]. These side effects may result from disruptions in the normal bacterial flora, direct toxic effects or chemical interactions with components of the host organism [10].

The number of bacteria, viruses, fungi, archaea, and protozoa that make up the gut microbiota in the gastrointestinal tract range from 10^10^ to 10^14^. Each species has co-evolved with this microbiota, which is essential for digestion and nutrient absorption, the production of short-chain fatty acids, amino acids, and vitamins, maintaining intestinal peristalsis and mucosal integrity, and the development of mucosal immunity [11]. Antibiotic use, however, can affect the diversity and stability of this microbiota, raising the risk of infections, causing the loss of good bacteria, allowing opportunistic pathogens to proliferate, and causing inflammation in the gut [12].

Given these potential systemic effects of antibiotics, Mus musculus—Swiss albino was chosen as the model organism to accurately assess these changes, due to its well-established role in biomedical research. The physiological and genetic similarities between mice and humans, along with their manageable size and reproductive characteristics, make them an ideal subject for studying the systemic effects of antibiotics [13]. Following the selection, the mice were administered the antibiotics orally. They were divided according to sex and treatment group, and were monitored for 14 days, with daily measurements of body weight, food consumption, and fecal output, while blood parameters were analyzed at the end of the study. The statistical data obtained offers a clear and convincing overview of the differences these antibiotics produce in healthy organisms.

The main aim of this study was to examine whether the oral administration of frequently used antibiotics induces systemic physiological and metabolic changes in healthy mice, and to find out whether these effects are mostly mediated indirectly via gut microbiota disruption or through direct interactions with host molecular targets. We expected that amoxicillin and tetracycline would variably influence body weight, dietary efficiency, and hematological markers, indicative of their unique antibacterial spectra and pharmacological characteristics. Furthermore, this study seeks to explore whether these antibiotics influence feeding behavior and metabolic processes, even in the absence of infection.

Beyond observing physiological changes, this study also aimed to understand how these antibiotics might interact with the host at a molecular level [14]. To explore this, we used molecular docking—a computational method that simulates how small molecules like drugs fit into protein targets in the body [15]. This approach helps predict whether antibiotics such as amoxicillin and tetracycline can bind directly to proteins involved in metabolism (like PPAR-γ) or immune function (like TLR2). Including molecular docking allows us to go beyond surface-level observations and ask whether the changes we see in weight, digestion, or immune markers could be linked to specific molecular interactions. By combining biological experiments with computational modeling, we gain a more complete picture of the potential side effects and mechanisms of action of these antibiotics, offering insight into how they may influence host physiology beyond their antimicrobial properties.

Based on the known effects of antibiotics on gut microbiota and host physiology, we hypothesized that oral administration of amoxicillin and tetracycline would induce systemic metabolic and immune alterations in healthy mice primarily through indirect microbiota-mediated mechanisms. Additionally, we hypothesized that tetracycline, due to its broader antimicrobial spectrum and greater electronic and structural flexibility, would exhibit stronger physiological and immunological effects than amoxicillin. As a secondary mechanistic objective, we further hypothesized that tetracycline may also directly interact with innate immune receptors such as TLR2, potentially contributing to immune modulation. To test these hypotheses, we combined in vivo physiological and hematological measurements with molecular docking and quantum chemical analyses to evaluate both indirect microbiota-mediated effects and the potential contribution of direct host receptor interactions.

## 2. Materials and Methods

In this study, thirty mice of the species Mus musculus—Swiss albino were used, weighing between 12 and 22 g and aged 6 to 8 weeks. The animals were randomly divided by sex, with 15 males and 15 females. They were housed in standard ventilated plastic cages, with five animals per cage. For each sex, the animals were further divided into three experimental groups: one group was treated with amoxicillin, another with tetracycline, and a third group was left untreated as a control. All animals were maintained under standard laboratory conditions with a 12 h light/dark cycle and ad libitum access to food and water throughout the study. To enable accurate collection of fecal matter, the cage bedding was removed and replaced with a clean cloth placed in one corner to ensure resting comfort.

The study commenced only after approval was obtained from the Subcommittee for Ethical Review of Research Involving Animals and Humans at the Faculty of Mathematical and Natural Sciences, University of Prishtina. The application for ethical approval was submitted on 16 April 2025 (Ref. No. 1219), and a positive recommendation was issued by the Ethics Committee for Research Involving Animals and Humans on 7 July 2025 (Ref. No. 2186). For this study, pharmaceutical-grade amoxicillin and tetracycline were administered, specifically ALMACIN^®^ 500 mg capsules (Alkaloid, Skopje, North Macedonia) and Tetraciclină Atb^®^ 250 mg capsules (Antibiotice SA, Bucharest, Romania).

Both antibiotics were prepared each day by dissolving the capsule powder in drinking water. Standard chow pellets were provided as food. Digital analytical scales and hemogram equipment were used for all measurements and analyses.

### 2.1. Methods

The treatment period of this research lasted for 14 consecutive days. Antibiotics were administered orally through drinking water, with dosages adjusted daily based on the average body weight per cage: 30 mg/kg/day for amoxicillin and 25 mg/kg/day for tetracycline. Water solutions were freshly prepared each morning and replaced every 24 h. Control animals were given only plain drinking water under the same conditions.

Each day 50 g of chow was placed in each cage, and the remaining food was measured after 24 h. The amount of food consumed daily was then calculated by subtracting the weight of the remaining food from the initial 50 g. Consumption per individual was also calculated by dividing by five. Feces were collected daily, air-dried at room temperature, and weighed. Daily body weight was recorded using a digital precision scale, always at the same hour to ensure consistency.

One methodological limitation of the present study is the absence of blinding during treatment administration and daily physiological measurements. Blinding was not performed during treatment administration due to the practical constraints of daily antibiotic preparation and cage-level dosing. However, hematological analyses were conducted using an automated system, minimizing operator-dependent bias.

Blood samples were collected at the end of the experimental period, via submandibular puncture using sterile lancets. Hematological analysis was performed using a standard automated hemogram system to measure white blood cell (WBC), red blood cell (RBC), hemoglobin (HGB), and platelet (PLT) levels.

### 2.2. Molecular Docking Methodology

Molecular docking was performed using the Schrödinger Maestro Suite (version 2024-1) to evaluate the binding interactions of amoxicillin and tetracycline with PPAR-γ (PDB ID: 3CS8) and TLR2–TIRAP (PDB ID: 5D3I). Protein structures were prepared using the Protein Preparation Wizard, which included removal of water molecules beyond 5 Å from heteroatoms, assignment of bond orders, addition of hydrogens, and restrained energy minimization using the OPLS4 force field. The chemical structures of amoxicillin and tetracycline were retrieved in SDF format from the PubChem database [16]. Ligands were prepared using LigPrep, generating ionization states at pH 7.0 ± 0.5 with the Epik module and energy-minimized conformers. Glide docking (Standard Precision, SP) was conducted using a grid box centered on the co-crystallized ligand or key binding residues, with default scaling of van der Waals radii (0.8) for nonpolar atoms. Binding free energies (ΔG_bind_) were calculated using the Prime MM-GBSA method with the VSGB 2.1 solvation model, providing a refined estimation of complex stability.

### 2.3. Statistics

Statistical analyses were conducted using Microsoft Excel and StatsKingdom software (https://www.statskingdom.com/). One-way ANOVA was used to compare means across groups, followed by Tukey’s HSD test where significance was found (*p* < 0.05). Results are expressed as mean ± standard error of the mean (SEM) for body weight measurements, and for hematological parameters, results are expressed as mean ± standard deviation (SD).

Additionally, the Food Efficiency Ratio (FER) was calculated by dividing the body weight gain by the amount of food consumed during the experimental period.

Data are reported separately for males and females, and are summarized using descriptive statistics, tables, and figures. Statistical significance was assessed using one-way ANOVA followed by Tukey’s HSD post hoc tests, with a threshold of *p* < 0.05. In tables and figures we used symbols * for *p* < 0.05, ** for *p* < 0.01 and *** for *p* < 0.001 to show the results significance.

## 3. Results and Discussion

The observed sex-specific differences in weight gain and food efficiency suggest that males and females may respond differently to antibiotic exposure at the physiological level. These differences could be related to known sex-dependent variations in hormone regulation, metabolic processes, and gut microbiota composition, all of which play important roles in maintaining metabolic and immune balance. In addition, the higher feces-to-food ratio observed in antibiotic-treated animals may reflect reduced digestive efficiency or impaired nutrient absorption, potentially as a result of antibiotic-induced disruption of the gut microbiota. However, because microbiota composition, intestinal function, and hormonal status were not directly measured in this study, these explanations should be considered as plausible interpretations rather than definitive conclusions. Further research incorporating microbiome sequencing, intestinal function assessments, and metabolic profiling will be necessary to clarify the precise mechanisms responsible for these physiological effects. The study aimed to investigate the systemic effects of two widely used antibiotics, amoxicillin and tetracycline, on feeding behavior, physiological parameters, and potential microbiota-related alterations in Mus musculus (Swiss albino).

Weight gain presented in Table 1 was lower in the antibiotic-treated groups in both male and female mice, compared to the control group. For male mice, the amoxicillin group showed a statistically significant reduction in weight gain compared to the control (*p* < 0.05), while the tetracycline group showed a highly significant reduction (*p* < 0.01). Similarly, in female mice, both the amoxicillin- and tetracycline-treated groups exhibited a highly significant decrease in weight gain compared to the control group (*p* < 0.01). These findings indicate that both antibiotics had an inhibitory effect on body weight gain in both sexes. Results are presented as mean ± standard error of the mean (SEM).

The decrease in body weight gain observed here also parallels findings by Galmiche et al. (2022) [17], who explain that infections and the immune response can lead to gut microbiota imbalances that affect appetite regulation. They suggest that even after the infection clears, these changes can cause long-term problems like reduced food intake, gut barrier disruption, and mood-related symptoms [17]. In our case, mice also showed signs of behavioral changes. They became unusually passive and showed very low activity levels in the antibiotic-treated groups.

**Figure 1:**
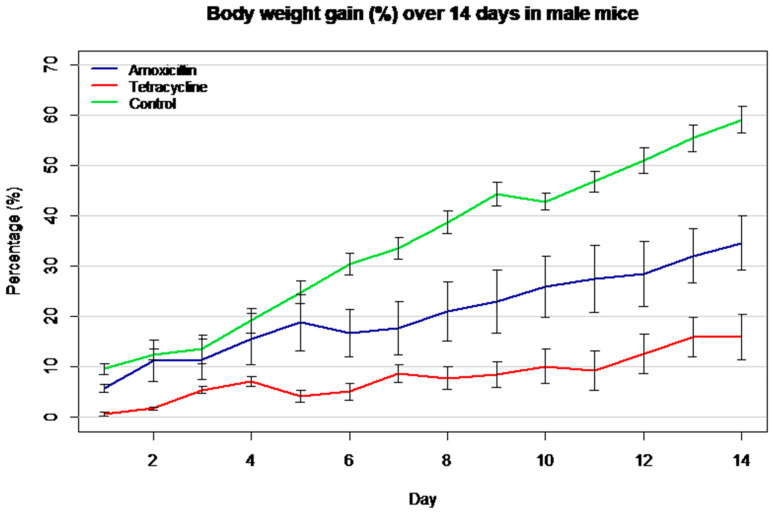
Body weight gain (%) over 14 days in male mice treated with antibiotics.

The graph (Figure 1) shows the percentage increase in body weight over a 14-day period in male Mus musculus (Swiss albino) mice. The control group exhibited the highest weight gain, followed by the amoxicillin group, while the tetracycline group showed the lowest increase. These trends indicate a negative impact of both antibiotics on growth performance, with tetracycline producing a more notable effect.

**Figure 2:**
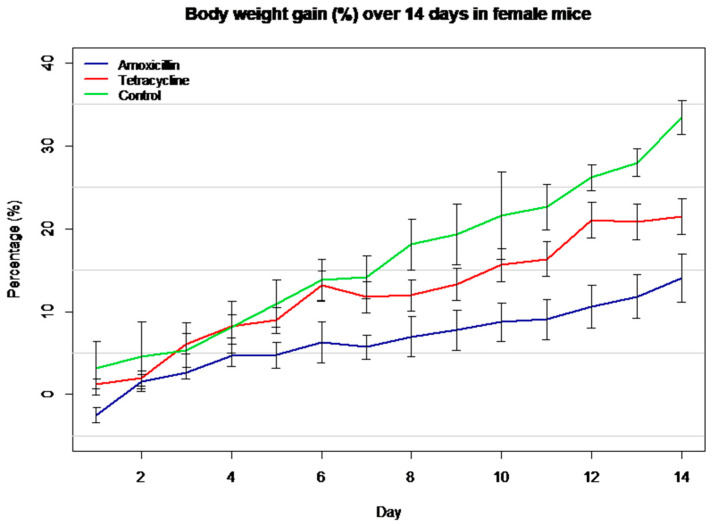
Body weight gain (%) over 14 days in female mice treated with antibiotics.

This graph (Figure 2) shows the percentage increase in body weight over a 14-day period in female mice. The control group gained the most weight, followed by tetracycline, while amoxicillin showed the lowest increase, indicating a stronger negative effect.

A recent systematic review by Sadighara et al. (2023) showed that even low doses of antibiotic residues in food can affect the gut microbiota, with tetracycline having one of the strongest effects and amoxicillin among the weakest [18]. In contrast, our findings in healthy mice indicate that both antibiotics caused noticeable changes in weight gain, food efficiency, and fecal output, especially with tetracycline in males and amoxicillin in females. While the review focused on long-term, low-level exposure, this study supports the idea that even short-term treatment with clinical doses can lead to clear metabolic and digestive changes, likely through microbiota disruption. This highlights the importance of monitoring both residue exposure and antibiotic use, even in healthy individuals.

**Figure 3:**
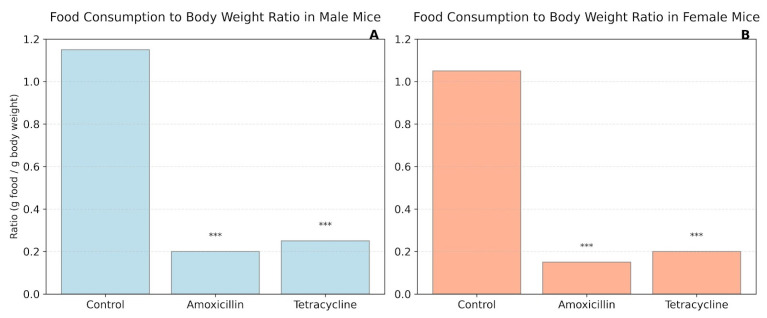
Food consumption to body weight ratio in mice. (**A**) Male mice; (**B**) Female mice. *** *p* < 0.001.

Figure 3 illustrates the food intake to body weight ratio across control and antibiotic-treated groups in both male (A) and female (B) mice. Administration of either amoxicillin or tetracycline resulted in a substantial and statistically significant reduction in the food-to-body weight ratio compared to untreated controls (*p* < 0.001) in both sexes. The ratios in antibiotic-treated animals were significantly lower, falling to roughly 0.19–0.24 in males and 0.15–0.19 in females, whereas control animals maintained similar ratios (1.15 for males and 1.04 for females).

**Figure 4:**
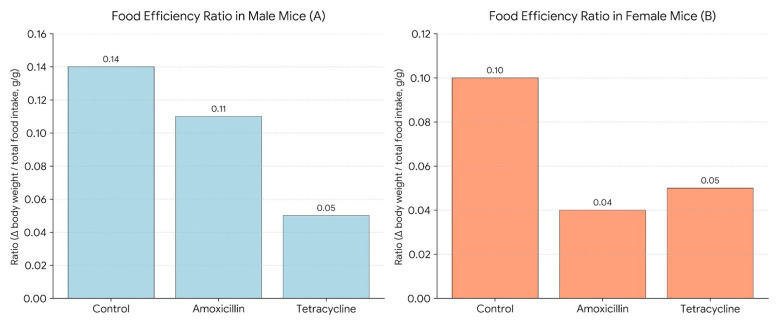
Feces-to-food ratio in male (**A**) and female (**B**) mice following 14-day antibiotic treatment.

Figure 4 shows the average feces-to-food ratio (g feces/g food consumed) in male and female mice after treatment with amoxicillin or tetracycline. In males (A), amoxicillin significantly increased the ratio compared to the control, whereas tetracycline did not cause a significant change. In females (B), both antibiotics significantly elevated the feces-to-food ratio compared to the control. In females, no significant differences were observed between the two antibiotic groups.

Food intake-to-body weight ratios were also significantly lower in both antibiotic-treated groups compared to controls. This could mean that the antibiotics affected digestion by altering the gut microbiome. Zimmermann and Curtis (2019) mention that gut dysbiosis caused by antibiotics can lead to mild gut discomfort, which might explain the lower food intake [19]. Another study in healthy human adults confirms that β-lactam antibiotics can disrupt gut microbiota. Since the gut microbiota is essential for digestion and nutrient absorption, this kind of dysbiosis could indirectly reduce feeding efficiency, which matches what we observed in our mice [20]. Also, Soares et al. (2021) found that tetracycline exposure changed the gut microbiota in bees, especially by reducing important bacteria involved in digestion and protection [21]. These changes can affect how well nutrients are absorbed and might explain the drop in food efficiency and body weight we saw in the treated mice.

Additionally, the feces-to-food ratio was also higher in the treated groups, suggesting inefficient digestion and absorption. This aligns with reports by Modi et al. (2014), who showed that antibiotic treatment can damage the intestinal mucosa and reduce absorptive surface area, leading to increased fecal output relative to intake [22].

**Figure 5:**
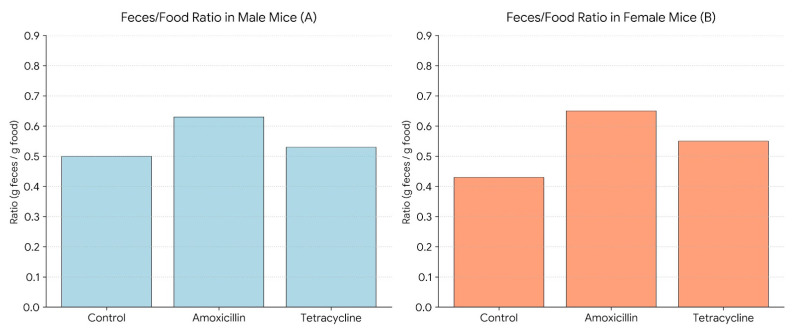
Food efficiency ratio in male (**A**) and female (**B**) mice.

Figure 5 illustrates the Food Efficiency Ratio (FER) in male (A) and female (B) mice. FER was calculated as the change in body weight divided by the total food intake over a 14-day period (Δ body weight/total food intake, g/g), providing a measure of how efficiently each group converted food into body mass. Antibiotic treatment reduced FER. As the graph shows in males, amoxicillin (0.11) and tetracycline (0.05) lowered efficiency compared to the control (0.14). In females, amoxicillin caused a sharp decline (0.04) from control (0.10), with tetracycline (0.05) showing slight improvement but still below control. These findings suggest antibiotics impair food efficiency, with a more pronounced effect in females, possibly due to sex-specific metabolic or microbiome differences.

The decrease in body weight was accompanied by reduced food efficiency ratios (FER), particularly in females treated with amoxicillin, which dropped to 0.04 compared to 0.10 in controls. This reduction can be explained by the way these antibiotics affect appetite, nutrient absorption, and overall metabolism. According to D’Alessandro et al. (2022) [23], penicillins like amoxicillin are linked to loss of appetite, tiredness, and weakness, all of which could have contributed to the lower food efficiency in the treated groups. Tetracycline, on the other hand, is known to interfere with the absorption of key nutrients such as iron and magnesium, which may impact both growth and blood parameters. These antibiotics may also alter the taste or smell of food—dysgeusia, defined as a distortion or alteration of taste perception—further reducing the animals’ interest in eating [23].

Table 2 shows the effects of amoxicillin and tetracycline on key blood parameters, such as white blood cells (WBCs), red blood cells (RBCs), hemoglobin (HGB), and platelets (PLTs). The male and female mice were compared to control groups. Significant changes in WBC counts are indicated by asterisks, reflecting the impact of antibiotics on immune and blood health. White blood cell counts were noticeably higher, especially in female mice, which suggests that disrupting the microbiota might have triggered some level of immune activation or inflammation throughout the body [23]. Interestingly, we had a significant drop in platelet counts (PLT) in male mice treated with tetracycline, which could suggest mild bone marrow suppression or changes in thrombopoiesis. Tetracyclines have been reported to negatively affect bone-related cellular activities, further supporting the possibility of bone marrow suppression, or altered thrombopoiesis [24].

This study showed that both antibiotics had a significant negative impact on growth, food efficiency, and blood parameters in healthy mice, with differences between males and females. The decrease in body weight gain was seen in both sexes, but tetracycline had a stronger effect in males, while amoxicillin caused more reduction in females. These findings support previous research that highlights the metabolic stress caused by antibiotics, even when there is no infection [25]. However, because gut microbiota composition and intestinal function were not directly measured, the proposed microbiota-mediated mechanisms should be interpreted cautiously and require further experimental confirmation.

### 3.1. Molecular Docking and Interaction Analysis of Antibiotics with PPAR-γ

**Figure 6:**
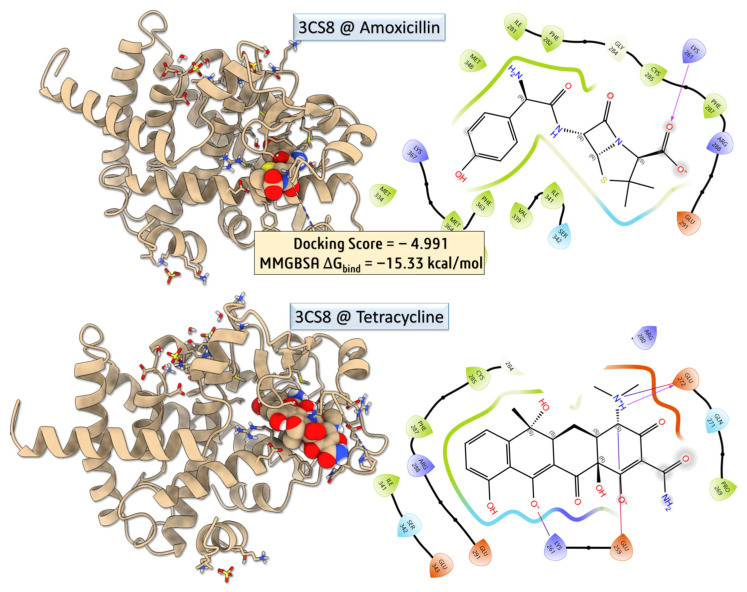
Molecular docking and MM-GBSA binding analysis of amoxicillin and tetracycline with the 3CS8 protein.

**Figure 7:**
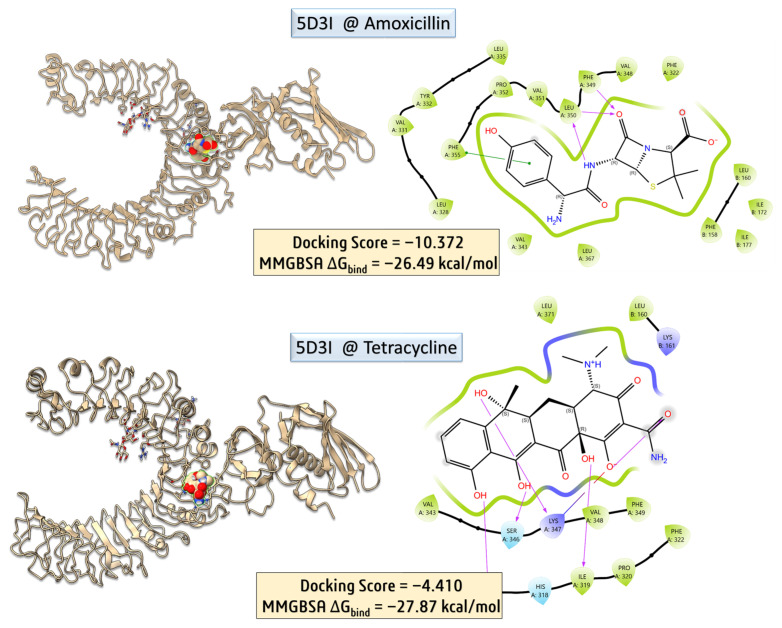
Molecular docking and MM-GBSA binding analysis of amoxicillin and tetracycline with the 5D3I protein.

To investigate the potential for direct modulation of host metabolic pathways by antibiotics, we performed molecular docking simulations (Figure 6 and Figure 7) of amoxicillin and tetracycline against the ligand-binding domain of PPAR-γ (PDB ID: 3CS8) [26]. This nuclear receptor plays a pivotal role in adipogenesis, lipid metabolism, and insulin sensitivity, and its activity can be modulated by endogenous ligands or pharmacological agents. The aim was to evaluate whether tetracycline, known for its ability to reduce body weight in mice, exhibits direct affinity for PPAR-γ that could contribute to such effects independently of microbiota disruption.

Despite a more favorable docking score for tetracycline, indicating strong spatial compatibility within the PPAR-γ binding pocket, its calculated MM-GBSA binding free energy was significantly less favorable than that of amoxicillin (−1.02 kcal/mol vs. −15.35 kcal/mol, respectively). This result suggests that although tetracycline fits well geometrically, it does not form a sufficiently stable complex to imply functional modulation of PPAR-γ.

Protein–ligand interaction profiling was conducted using the Protein–Ligand Interaction Profiler (PLIP) [27], which revealed key non-covalent interactions, including hydrogen bonds, π–π stacking, and hydrophobic contacts, supporting the observed differences in binding stability between the two ligands. Amoxicillin–PPAR-γ Complex—Amoxicillin exhibited a moderate docking pose stabilized by the hydrophobic contacts with PHE287 (3.43 Å) and ARG288 (3.85 Å), contributing to van der Waals stabilization, and the salt bridge with LYS261 (3.24 Å), involving its carboxylate group, provides strong electrostatic anchoring within the binding cleft. Despite the absence of hydrogen bonds, the low MM-GBSA binding free energy suggests that electrostatic and hydrophobic contacts are sufficient to confer substantial binding affinity. Nevertheless, amoxicillin does not induce metabolic alterations such as weight loss, indicating that this binding is likely pharmacologically irrelevant in vivo. Tetracycline–PPAR-γ Complex—Tetracycline exhibited a rich interaction profile, comprising five hydrogen bonds, notably with LYS261, SER342, ILE281, and PRO269, primarily involving backbone amide groups and polar functional moieties. Additionally, it formed three hydrophobic contacts with ILE341, PHE287, and ARG288, contributing to van der Waals stabilization within the binding pocket. The interaction network also included two salt bridges: one with LYS261 (4.22 Å), which was weaker compared to that observed in the amoxicillin complex, and another with GLU272 (3.71 Å), involving the amine functionalities of tetracycline. These interactions suggest a more complex binding mode, involving both polar and nonpolar contacts, consistent with its polyfunctional structure [28]. However, the relatively high MM-GBSA binding energy (−1.02 kcal/mol) indicates that the binding may be transient or energetically unfavorable, limiting its likelihood of affecting receptor function [29]. While tetracycline displayed a more favorable docking score and a denser interaction network with PPAR-γ, its weak MM-GBSA ΔG_bind_ value suggests that this binding is unlikely to be biologically relevant. Given that amoxicillin, which showed stronger binding energetics, does not induce weight loss, and tetracycline’s effects on body weight are well-established through gut microbiota depletion, the results support the hypothesis that tetracycline’s metabolic effects are indirect, rather than due to direct PPAR-γ engagement. This aligns with earlier studies showing that tetracycline disrupts the production of microbial short-chain fatty acids (SCFAs) and upregulates ANGPTL4, leading to suppressed lipoprotein lipase (LPL) activity and reduced lipid accumulation [29]. The lack of strong binding to PPAR-γ further emphasizes the importance of microbiota-mediated host signaling in antibiotic-induced metabolic alterations.

To further elucidate potential immunomodulatory effects of antibiotic treatment, molecular docking studies were extended to the Toll-like receptor 2 (TLR2) signaling complex, using the crystal structure of the human TLR2–TIRAP complex (PDB ID: 5D3I) [30]. This receptor is a key player in the innate immune system, recognizing bacterial lipoproteins and modulating inflammatory responses. Amoxicillin and tetracycline were docked into the putative ligand-binding region of TLR2. While tetracycline showed a moderately favorable docking score and interaction with residues involved in signal propagation, amoxicillin exhibited weaker binding affinity and fewer stabilizing interactions. These results suggest that tetracycline may more plausibly interfere with TLR2-mediated immune signaling, potentially contributing to observed alterations in immune cell counts or inflammatory responses post-treatment.

Molecular docking and MM-GBSA calculations were carried out to assess the binding potential of amoxicillin with the human TLR2–TIRAP complex (PDB ID: 5D3I). The docking yielded a highly favorable score of −10.372, with an MM-GBSA binding free energy (ΔG_bind_) of −26.49 kcal/mol, indicating a strong and stable interaction. PLIP interaction profiling revealed multiple stabilizing contacts, including five hydrophobic interactions with residues ILE177 (chain B), LEU328, VAL331, LEU350, and PRO352 (chain A), as well as three hydrogen bonds involving PHE349 and LEU350. Notably, π–π stacking was observed between amoxicillin’s aromatic moiety and PHE355, with a centroid-to-centroid distance of 4.02 Å and a favorable angle of 21.13°, suggesting significant stacking contribution to binding affinity. These interactions collectively reinforce the strong binding energy obtained, supporting the hypothesis that amoxicillin may interact with immune receptors such as TLR2, potentially modulating downstream immune responses.

To evaluate the binding characteristics of tetracycline with the TLR2–TIRAP complex, detailed post-docking interaction profiling was performed using the Protein–Ligand Interaction Profiler (PLIP). The docking simulation yielded a moderate docking score of –4.410, yet the MM-GBSA binding free energy (ΔG_bind_) was notably favorable at −27.87 kcal/mol, indicative of a stable and energetically preferred complex formation.

PLIP analysis revealed an extensive network of non-covalent interactions involving both hydrophobic and polar residues across chains A and B. A total of three hydrophobic interactions were identified, predominantly involving PHE322 and PHE349 in chain A. These residues engaged in close van der Waals contacts (3.41–3.54 Å) with the tetracycline aromatic scaffold, suggesting π-alkyl or edge-to-face π–π stabilization within a semi-aromatic binding pocket. Additionally, eight hydrogen bonds were detected, forming a robust and spatially distributed polar interaction network. These included interactions with LYS161 (chain B), which contributed a strong charge-assisted hydrogen bond (donor–acceptor distance: 2.89 Å; donor angle: 147.3°), stabilizing the interfacial region between the two chains. On chain A, key hydrogen bonds involved LEU317, ILE319, PRO320, PHE322, SER346, and LYS347. Notably, LYS347 participated in dual hydrogen bonding events, acting as both a donor (via its ε-amino group) and an acceptor (via backbone carbonyl), providing bidirectional stabilization to the ligand. The ligand also formed an additional hydrogen bond with PHE322, suggesting cooperative interactions that strengthen binding at this site. Collectively, these findings support the notion that tetracycline engages the TLR2–TIRAP complex through a combination of hydrophobic interactions and an extensive hydrogen bonding network. This interaction profile aligns well with the calculated MM-GBSA energy and supports a mechanistic hypothesis wherein tetracycline may directly modulate innate immune signaling through engagement with TLR2. Further experimental validation, such as site-directed mutagenesis or cellular reporter assays, would be necessary to confirm the functional relevance of this predicted binding mode.

Importantly, the computational findings help explain the immune-related changes observed in vivo. Tetracycline demonstrated strong binding affinity to the TLR2 receptor, supported by favorable MM-GBSA binding energy and extensive hydrogen bonding interactions. Since TLR2 is a key mediator of innate immune responses, this interaction may contribute to the observed alterations in white blood cell counts and immune parameters in treated mice. Furthermore, DFT and QTAIM analyses revealed that tetracycline possesses greater electronic softness, higher electrophilicity, and an extensive hydrogen-bonding network, which enhance its ability to interact with protein targets. These electronic and structural features provide a mechanistic explanation for its stronger predicted interaction with immune receptors compared to amoxicillin. Together, these computational and experimental findings suggest that antibiotic-induced physiological effects may result not only from microbiota disruption but also from potential modulation of host immune signaling pathways.

### 3.2. Frontier Molecular Orbital Analysis and Global Reactivity Descriptors

The electronic characteristics of amoxicillin and tetracycline were investigated using frontier molecular orbital (FMO) theory and global reactivity descriptors derived from density functional theory (DFT). All calculations were performed at the ωB97XD/6-311+G(d,p) level of theory [31] with an implicit water solvent model (IEFPCM) [32]. These descriptors provide essential insights into the reactivity, chemical stability, and potential interaction mechanisms of the two antibiotics when targeting biological macromolecules.

#### Frontier Orbital Energies and HOMO–LUMO Gap

**Figure 8:**
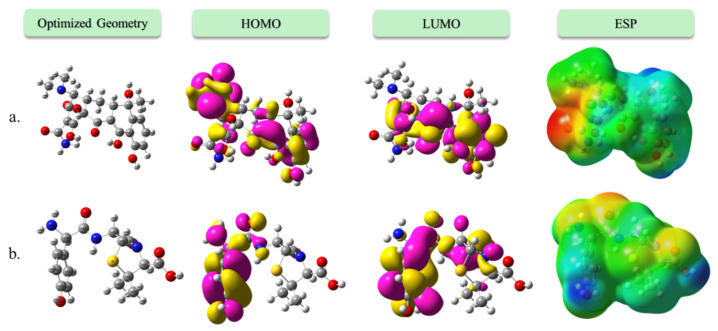
Optimized geometries, frontier molecular orbitals (HOMO and LUMO), and electrostatic potential (ESP) maps of amoxicillin (**a**) and tetracycline (**b**). HOMO and LUMO isosurfaces highlight the localization of electron-donating and accepting regions, respectively.

The HOMO and LUMO energies and the corresponding energy gaps are summarized in Table 3. Amoxicillin exhibits a HOMO energy of −8.377 eV and a LUMO energy of +1.014 eV, resulting in a HOMO–LUMO energy gap of 9.39 eV (Figure 8). In contrast, tetracycline presents a HOMO energy of −8.343 eV and a LUMO energy of −0.935 eV, yielding a smaller energy gap of 7.41 eV. The significantly larger gap in amoxicillin implies greater chemical stability and lower polarizability, characteristics that correlate with its highly localized mode of reactivity, particularly in covalent interactions with serine residues of penicillin-binding proteins [33].

On the other hand, the narrower gap in tetracycline suggests increased electronic softness and greater susceptibility to charge redistribution, which is advantageous for reversible binding, metal chelation, and π-stacking interactions, consistent with its mode of action.

### 3.3. Global Reactivity Descriptors

Using Koopmans’ approximation, key global descriptors [33,34]—ionization potential (IP), electron affinity (EA), chemical potential (μ), chemical hardness (η), softness (S), and electrophilicity index (ω)—were computed and are presented in Table 3.

Amoxicillin’s higher chemical hardness (η = 4.70 eV) and lower electrophilicity index (ω = 1.44 eV) are consistent with its rigid, site-specific interactions and reduced charge transfer capacity. Tetracycline, on the other hand, is characterized by a greater softness and a higher electrophilicity index, confirming its greater adaptability in forming noncovalent interactions, particularly with metal ions or nucleobase sites.

The contrasting electronic profiles of amoxicillin and tetracycline underscore their distinct biological behavior. Amoxicillin functions as a classical electrophilic agent, with a strongly localized HOMO on the β-lactam moiety and large band gap that limits nonspecific interactions. Its high hardness supports its role in irreversible covalent inhibition of serine-based enzymes. Tetracycline, due to its extended conjugated system and delocalized frontier orbitals, displays a softer, more electrophilic nature. Its LUMO is lower in energy (−0.94 eV), favoring charge-transfer complexes with metal cofactors or interaction with π-rich environments in protein targets. These findings provide a mechanistic basis for the selective binding and pharmacological diversity of these antibiotics, complementing the docking simulations and experimental binding studies.

### 3.4. Population Analysis and Electrostatic Characteristics of Amoxicillin and Tetracycline

**Figure 9:**
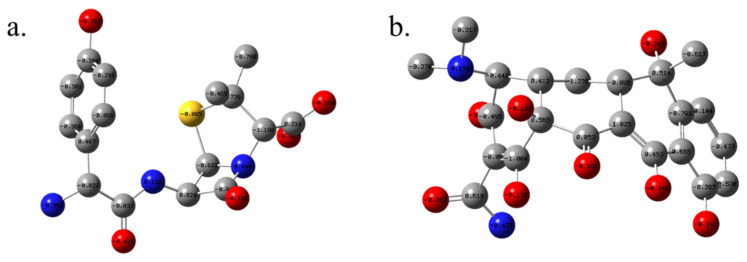
Mulliken atomic charges for optimized structures of amoxicillin (**a**) and tetracycline (**b**), calculated at the ωB97XD/6-311+G(d,p) level in water using the IEFPCM. (hydrogen atoms are omitted for clarity).

Mulliken Charge Distribution (MCD): Mulliken population analysis was employed to assess the electron density distribution and to identify chemically reactive centers relevant to noncovalent interactions during molecular docking [35]. Amoxicillin exhibits a highly polarized electronic structure. The MCD (Figure 9) shows that the oxygen atoms, particularly O6 (−0.331), O3 (−0.256), and O5 (−0.254), carry substantial negative charge, making them favorable hydrogen bond acceptors. The most negatively charged atom is N9 (−0.546), situated within the β-lactam framework, indicating a strong nucleophilic center. In contrast, C20 (+1.163) represents a highly electropositive site, likely participating in cation–π or polar interactions.

The carbon atom C22 (−1.148) is notably electron-rich and may engage in π–π stacking or act as a chelation site. In tetracycline, a different polarity pattern emerges due to its extended π-conjugated system. The most negatively charged atoms include O8 (−0.380), O7 (−0.248), and O6 (−0.204), all located on hydroxyl or carbonyl groups. N10 (−0.543), similar to amoxicillin’s N9, appears as a key nucleophilic site. Significantly negative carbons include C13 (−1.266), C21 (−1.023), and C12 (−0.838), localized in conjugated aromatic and enol systems, reinforcing tetracycline’s ability to engage in π–π stacking, resonance-assisted hydrogen bonding, and metal ion chelation. C30 (+0.575) and C11 (+0.485) mark electrophilic regions likely to receive nucleophilic attack.

Hirshfeld and CM5 Charges: To provide a more physically grounded perspective, Hirshfeld and CM5 charge analyses were also performed. In amoxicillin, Hirshfeld charges [36] confirm the high electron density at O6 (−0.227) and N9 (−0.223), while CM5 adjustments further polarize these atoms (e.g., O6: −0.423, N9: −0.679), consistent with their hydrogen bonding potential. Similarly, C22 maintains a high negative CM5 charge [37] supporting its likely participation in electrostatic or donor–acceptor interactions in enzyme binding pockets. For tetracycline, Hirshfeld analysis identifies O8 (−0.361) and O6 (−0.278) as the most nucleophilic centers, closely mirrored in CM5 values (O8: −0.399, O6: −0.327). The CM5 charges on N10 (−0.570) and N9 (−0.372) confirm a strong lone-pair character ideal for coordination with metal ions or acceptor groups in active sites.

### 3.5. Dipole Moment and Spatial Extent

The total dipole moment of amoxicillin is 3.29 Debye, with significant components along all axes, indicating moderate polarity in aqueous environments and potential for multi-directional electrostatic interactions.

By contrast, tetracycline exhibits a significantly higher dipole moment of 10.27 Debye, driven primarily by its extended π-conjugation and polar substituents. This large dipole is consistent with its amphoteric character, suggesting strong solvation effects and directional binding affinity in docking scenarios. The population analyses highlight distinct reactivity patterns: Amoxicillin tends to bind via localized polar sites, especially through the β-lactam ring and adjacent carboxylate moiety. These features support its specific interactions with serine residues or backbone carbonyls in penicillin-binding proteins. Whereas, tetracycline, on the other hand, offers a delocalized electron framework and multiple overlapping nucleophilic regions, which are advantageous for bidentate chelation, π-stacking, and metal-mediated docking in ribosomal or metalloenzyme targets. The combined data from Mulliken, Hirshfeld, and CM5 approaches provide robust electrostatic mapping to rationalize binding preferences observed in docking simulations, and underscore the chemical complementarity between antibiotic structure and biological macromolecular surfaces.

### 3.6. Topological Analysis of Electron Density: QTAIM Perspective on Amoxicillin and Tetracycline

**Figure 10:**
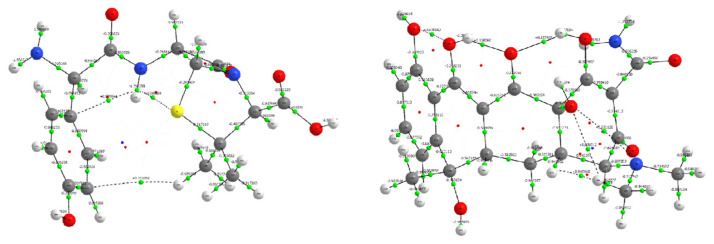
Molecular graphs generated from QTAIM analysis for amoxicillin (**left**) and tetracycline (**right**). Bond critical points (green spheres), and bond paths (white lines) are displayed over the molecular structures. Atom color coding: carbon (gray), hydrogen (white), oxygen (red), nitrogen (blue), and sulfur (yellow, amoxicillin only).

To elucidate the electronic structure and bonding features of the antibiotic molecules amoxicillin and tetracycline, a comprehensive Quantum Theory of Atoms in Molecules (QTAIM) [38] analysis was performed (Figure 10). Topological parameters including electron density at the bond critical point (ρ), and its Laplacian (∇^2^ρ), ellipticity (ε), and local energy densities (kinetic G, potential V, and total energy density H) were used to evaluate the bonding character and identify potential noncovalent interactions.

Covalent Bonding Framework: Both molecules display strong shared-shell covalent interactions across their backbone C–C and C–H bonds. In amoxicillin, BCPs such as C12–C17 (ρ = 0.258 a.u., ∇^2^ρ = −0.647 a.u.) and C11–C12 (ρ = 0.227 a.u.) exhibit high electron densities and negative Laplacians, affirming classical covalent bond characteristics. Likewise, tetracycline shows consistent features with ρ ranging from 0.24 to 0.31 a.u. and ∇^2^ρ < 0 for most ring and aliphatic C–C and C–H bonds (e.g., C20–C26, C31–C32), confirming strong covalent character. Low ellipticity values (ε < 0.05) in many single bonds across both structures indicate a predominant σ-character [39]. However, in tetracycline, certain conjugated systems (e.g., C19–C23, ε = 0.388) exhibit elevated ellipticity, suggesting π-delocalization within its polycyclic ring system—an important feature for molecular recognition and stacking interactions.

Polar Covalent and Highly Polar Bonds: In both antibiotics, heteroatomic bonds such as C–N, C–O, and C–S exhibit increased polarity. For example, in amoxicillin, the C18–O5 bond (ρ = 0.401 a.u., ∇^2^ρ = −0.206 a.u., H = −1.289 a.u.) and C17–O4 (ρ = 0.418 a.u., H = −1.403 a.u.) represent strong, localized polar covalent bonds. In tetracycline, similar features are observed in O3–C18 (ρ = 0.367 a.u., H = −0.212 a.u.) and O8–C30 (ρ = 0.401 a.u., H = −0.255 a.u.), suggesting significant electron accumulation on oxygen atoms, which may act as hydrogen bond acceptors or metal-binding sites [40]. The higher potential energy densities (V) and large absolute H values in these bonds support their electrophilic or nucleophilic roles, depending on context.

Intramolecular Hydrogen Bonding and Electrostatic Interactions: Several weak interactions were identified that resemble intramolecular hydrogen bonds. In amoxicillin, BCPs such as S1–H35 (ρ = 0.015 a.u., ∇^2^ρ = +0.050 a.u., H = −0.009 a.u.) and O6–H44 (ρ = 0.367 a.u., ∇^2^ρ = +2.623 a.u., H = −0.790 a.u.) suggest both weak and moderately strong hydrogen bonding [41]. These are essential in stabilizing the bioactive conformations of the drug. In tetracycline, similar behavior is observed: BCPs like O5–H51 (ρ = 0.335 a.u., ∇^2^ρ = +2.389 a.u.) and O7–H56 (ρ = 0.367 a.u., ∇^2^ρ = +2.627 a.u.) represent strong hydrogen bonding typical of hydroxyl and enolic functionalities, stabilizing the polycyclic skeleton through intramolecular H-bond networks. Their high G/|V| ratios and negative total energy densities confirm a mixed electrostatic-covalent character, especially relevant to the molecule’s acid-base behavior and solvation.

Conjugation and Delocalized π-Systems: Ellipticity analysis reveals significant π-delocalization in tetracycline, particularly across the aromatic and enone systems. BCPs such as C19–C23 (ε = 0.388) and C24–C29 (ε = 0.252) indicate bond path curvature and electron delocalization consistent with resonance-stabilized systems. These regions may be involved in stacking interactions and are often the target of DNA intercalation or protein binding in antibiotics. Amoxicillin shows more limited π-character, with moderately elevated ellipticity observed in bonds like N8–C18 (ε = 0.096), consistent with localized lone-pair interactions rather than extended conjugation.

The QTAIM analysis underscores the electronic and structural differences between amoxicillin and tetracycline, reflecting their distinct pharmacophores and biological activities. While amoxicillin exhibits well-defined σ-bonding and localized polar interactions characteristic of β-lactam antibiotics, tetracycline presents extensive conjugation, π-delocalization, and a robust hydrogen bonding network, aligning with its polyfunctional, multi-target binding profile. These insights are essential for understanding drug–target interactions, predicting reactivity, and optimizing molecular design in antibiotic development.

## 4. Conclusions

This study shows that the commonly used antibiotics amoxicillin and tetracycline can affect the body in more ways than just fighting infections. When given to healthy mice, both drugs led to noticeable changes in weight gain, food efficiency, and blood cell counts, even without any underlying illness. These effects were different for males and females, with tetracycline reducing weight more strongly in males, while amoxicillin had a bigger impact on food efficiency in females. To understand why this happens, we used molecular docking simulations to see whether these antibiotics interact with key proteins in the body. We tested two types of proteins: PPAR-γ, which helps regulate fat and metabolism, and TLR2, which plays a major role in the immune system. The results showed that neither antibiotic formed a strong or stable connection with PPAR-γ, suggesting that the metabolic changes we observed are not due to direct effects on this receptor. However, tetracycline showed strong and stable interactions with TLR2, which may help explain the immune-related changes we saw in the blood. Overall, our findings suggest that antibiotics like amoxicillin and tetracycline can disrupt normal body processes—possibly by affecting gut bacteria or immune signaling—even when no infection is present. This highlights the need for more cautious and targeted use of antibiotics, especially when treating healthy individuals or using them preventively.

The current study has certain limitations that we recognize. Initially, while the noted physiological and hematological alterations strongly indicate microbiota-mediated consequences of antibiotic treatment, direct assessments of gut microbiota composition (such as 16S rRNA sequencing or metagenomic profiling) were not conducted. As a result, judgments on microbiome disruption are derived rather than empirically validated. The use of healthy mice without underlying illnesses limits the applicability of the findings to clinical or pathological contexts, where host–microbiota–immune interactions may differ considerably. Nonetheless, using healthy animals offers direct insight into the unintended consequences of antibiotic exposure on the normal gut microbiota, facilitating the evaluation of physiological and metabolic changes without disease-related confounding variables. However, further studies incorporating direct microbiome analysis and functional validation are required to fully confirm the mechanistic pathways proposed in this study.

## Figures and Tables

**Table 1:** Body weight changes in male and female mice during the experimental period (mean ± SEM).

Sex	Group	Initial Weight (g)	Final Weight (g)	Weight Gain (g)
Male	Control	12.88 ± 0.48	20.53 ± 0.97	7.65 ± 0.54
	Amoxicillin	14.92 ± 0.89	20.12 ± 1.49	5.20 ± 0.83 *
	Tetracycline	12.95 ± 0.61	15.07 ± 1.38	2.12 ± 1.02 **
Female	Control	15.95 ± 0.74	21.25 ± 0.76	5.31 ± 0.19
	Amoxicillin	20.68 ± 0.32	22.66 ± 0.64	1.98 ± 0.33 **
	Tetracycline	19.59 ± 1.07	22.37 ± 0.90	2.78 ± 0.45 **

* *p* < 0.05; ** *p* < 0.01.

**Table 2:** Hematological parameters in male and female mice after antibiotic treatment.

Sex	Group	WBC (10^3^/µL)	RBC (10^6^/µL)	HGB (g/dL)	PLT (10^3^/µL)
Male	Control	9.31 ± 0.29	9.87 ± 1.28	13.7 ± 1.25	344 ± 35.66
	Amoxicillin	7.75 ± 0.68 **	10.37 ± 0.69	14.72 ± 0.97	341.6 ± 29.77
	Tetracycline	10.53 ± 0.50 *	8.93 ± 1.19	12.02 ± 1.49	252.75 ± 37.80
Female	Control	5.66 ± 0.57	9.96 ± 0.94	14.3 ± 1.30	267.67 ± 40.89
	Amoxicillin	9.47 ± 1.32 *	10.88 ± 0.42	15.52 ± 0.46	369.33 ± 39.90
	Tetracycline	8.55 ± 0.95 *	10.55 ± 0.42	14.57 ± 0.46	430.33 ± 33.94

* *p* < 0.05; ** *p* < 0.01.

**Table 3:** Frontier molecular orbital energies and DFT-derived global reactivity descriptors for amoxicillin and tetracycline calculated at the ωB97XD/6-311+G(d,p) level with IEFPCM (water) solvent model.

Descriptor	Amoxicillin	Tetracycline
HOMO (eV)	−8.377	−8.343
LUMO (eV)	+1.014	−0.935
HOMO–LUMO Gap (eV)	9.391	7.407
Ionization Potential (IP, eV)	8.377	8.343
Electron Affinity (EA, eV)	−1.014	0.935
Chemical Potential (μ, eV)	−3.682	−3.704
Chemical Hardness (η, eV)	4.696	3.704
Chemical Softness (S, eV^−1^)	0.213	0.270
Electrophilicity Index (ω, eV)	1.44	1.85

## Data Availability

The data associated with this manuscript are partly included in the article; additional data are available from the corresponding author upon reasonable request.
